# Landscape of transcriptional deregulation in lung cancer

**DOI:** 10.1186/s12864-018-4828-1

**Published:** 2018-06-05

**Authors:** Shu Zhang, Mingfa Li, Hongbin Ji, Zhaoyuan Fang

**Affiliations:** 1 School of Life Sciences and Biotechnology, Shanghai Jiao Tong University, Shanghai, 200240 People’s Republic of China; 2 State Key Laboratory of Cell Biology, Shanghai, China; 3 CAS Center for Excellence in Molecular Cell Science, Shanghai, China; 4 Innovation Center for Cell Signaling Network, Institute of Biochemistry and Cell Biology, Shanghai, 200031 China; 5 School of Life Science and Technology, Shanghai Tech University, Shanghai, 200120 China; 6 Shanghai Institutes for Biological Sciences, Chinese Academy of Science, Shanghai, 200031 China

**Keywords:** Lung cancer, Transcription factors, Support-vector machines, Transcription regulatory network

## Abstract

**Background:**

Lung cancer is a very heterogeneous disease that can be pathologically classified into different subtypes including small-cell lung carcinoma (SCLC), lung adenocarcinoma (LUAD), lung squamous cell carcinoma (LUSC) and large-cell carcinoma (LCC). Although much progress has been made towards the oncogenic mechanism of each subtype, transcriptional circuits mediating the upstream signaling pathways and downstream functional consequences remain to be systematically studied.

**Results:**

Here we trained a one-class support vector machine (OC-SVM) model to establish a general transcription factor (TF) regulatory network containing 325 TFs and 18724 target genes. We then applied this network to lung cancer subtypes and identified those deregulated TFs and downstream targets. We found that the TP63/SOX2/DMRT3 module was specific to LUSC, corresponding to squamous epithelial differentiation and/or survival. Moreover, the LEF1/MSC module was specifically activated in LUAD and likely to confer epithelial-to-mesenchymal transition, known important for cancer malignant progression and metastasis. The proneural factor, ASCL1, was specifically up-regulated in SCLC which is known to have a neuroendocrine phenotype. Also, ID2 was differentially regulated between SCLC and LUSC, with its up-regulation in SCLC linking to energy supply for fast mitosis and its down-regulation in LUSC linking to the attenuation of immune response. We further described the landscape of TF regulation among the three major subtypes of lung cancer, highlighting their functional commonalities and specificities.

**Conclusions:**

Our approach uncovered the landscape of transcriptional deregulation in lung cancer, and provided a useful resource of TF regulatory network for future studies.

**Electronic supplementary material:**

The online version of this article (10.1186/s12864-018-4828-1) contains supplementary material, which is available to authorized users.

## Background

Lung cancer is the leading cause of cancer-related deaths worldwide. Pathologically, lung cancers can be classified as small-cell lung carcinoma (SCLC) and non-small-cell lung carcinoma (NSCLC), and the latter can be further divided into lung adenocarcinoma (LUAD), lung squamous cell carcinoma (LUSC), and others such as large-cell carcinoma (LCC). Among these lung cancer subtypes, LUAD, LUSC and SCLC are most prevalent, accounting for about 40%, 25-30% and 10-15% respectively (https://www.cancer.org). Previous mechanistic studies have greatly advanced our knowledge about how lung cancer initiates, progresses and responds to drug treatments [1–3]. However, it remains interesting to systematically uncover the molecular regulatory network in contributing to lung cancer malignant progression.

Transcription factors (TFs), known to be evolutionarily conserved in orchestrating transcriptional gene regulation networks, are the key players in contribution to a broad range of critical cellular physiological and pathological processes, from normal development and physiological processes to diseases such as cancer [4–7]. Notably, master TFs bind to the corresponding promoter regions via recognizing specific short sequence patterns (‘motifs’), and regulate transcriptional expression of a series of target genes, which thus control cell growth, proliferation and differentiation. For instance, TFs such as PPARγ and C/EBPα are key regulators of adipogenic differentiation [8]. Overexpression of TFs including OCT4, SOX2, KLF4 and MYC can reprogram fibroblasts to pluripotent stem cells [9, 10]. Nanog, another TF which is transcriptionally regulated by OCT4 and SOX2, is also important for the maintenance of pluripotency [11]. Furthermore, TFs are the major driving forces of transdifferentiation and transition among different cell types [12]. Such TF regulatory programs also exist in cancer. For example, the epithelial-to-mesenchymal transition (EMT) process, mediated by key TFs such as SNAILs and bHLHs, is known to promote cancer malignant progression and metastasis [13, 14]. The reprogramming factor, SOX2, has also been identified as a lineage-survival oncogene in LUSC [15]. SOX2 and TP63 (the other known LUSC lineage TF) are both frequently amplified and crucial for LUSC development [15–17]. Recently, we have also shown that, TP63 mediates the transdifferentiation from LUAD to LUSC [18].

To systematically understand how transcription factors contribute to the malignant progression of lung cancer, we employed a machine learning approach to build a transcriptional regulatory network, based on curated regulatory relations, motif distributions, protein-protein interactions (PPIs) and gene co-expression. With the application of this network in LUSC, LUAD and SCLC, we identified those core TFs specific for each lung cancer subtype. We further described the landscape of TF deregulation in these three major lung cancer subtypes.

## Methods

### Lung cancer data sources and preprocessing

The RNA-Seq FPKM and copy number data for TCGA LUAD and LUSC were downloaded from the UCSC Xena hub (http://xena.ucsc.edu/). The SCLC gene expression data were obtained from the paper-accompanied data [19]. Other LUAD and LUSC data outside of TCGA were downloaded from the NCBI GEO with accession number GSE81089. To be concise, we refer to these LUAD and LUSC datasets outside of TCGA as ‘LUAD2’ and ‘LUSC2’. For FPKM data, a log-transformation was applied before downstream analyses of co-expression and differential expression.

### Promoter sequences and motif analyses

We obtained genomic sequences (UCSC hg19) from 10kb upstream to 10kb downstream of TSS for each Ensembl gene. Non-redundant TF motifs were from the JASPAR database [20] and converted to MEME format. Additional motifs (NKX2-1 and ASCL1) were trained from the reported TF binding peaks [21, 22], with the MEME-ChIP pipeline [23]. Scanning of motifs along promoter sequences was performed with FIMO (default *p* value threshold, 1e-4) [24]. FIMO matches on each strand were categorized by upstream 10kb, 2kb, 500b and downstream 10kb, 2kb, 500b, respectively.

### Gene co-expression and network neighborhood analyses

We downloaded the comprehensive tissue profiling data from the GTEx project (version v6p) [25]. After logarithmic transformation and quantile normalization with voom [26], Pearson Correlation Coefficient (PCC) was computed for each pair of genes. Protein-protein interactions were downloaded from the integrated EBI IntAct molecular interaction database [27]. For each candidate gene, its PCCs with the TF and TF-interacting proteins (‘neighbors’) were computed, and the latter PCCs were summarized into three quantiles (25% as Q1, 50% as M, 75% as Q3). The candidate gene’s PCCs with the background genes were also calculated and summarized into these three quantiles.

### OC-SVM model training and evaluation

One-class support vector machine (OC-SVM) is a special type of SVM model suitable for solving problems where high-quality training data is available for only one class, and it has been widely used in single-class learning and outlier detection [28, 29]. Here we used curated TF-target relations from the TRRUST database as the positive training set [30], with synthetic negatives to evaluate the model performance. The negative set was built with 1000 20kb random sequences scanned with FIMO using the same setting. The correlation coefficient data for synthetic genes were randomly chosen from real gene correlation coefficients. A random subset of 50,000 TF-target pairs were used for evaluation. The OC-SVM model was trained using the libSVM R wrapper in the e1071 package. With the radial basis kernel and a series of ‘nu’ (ranging between 1^-4 and 0.9) and ‘gamma’ (2^-5, 2^-8, 2^-11), the performance of models were assessed in terms of sensitivity and false positive rate (FPR) with 10-fold cross-validation. To achieve a high specificity that is essential for large-scale predictions where the candidate relations are huge (over 17,000,000), we controlled the final model (nu=0.5, gamma=2^-5) at a relatively low FPR (0.002), sacrificing some sensitivity (50%). This predicted 2,432,769 relationships between TFs and protein-coding target genes, and ~5000 of them were likely to be false positives.

### Identification of core TFs in lung cancer

To ensure specificity on the lung cancer dataset, we filtered the predicted targets for individual TFs by enforcing two sequential steps: (i) the target gene must have conditional co-expression with the TF (PCC>=0.5); (ii) the target gene must have inter-correlations with at least 1/6 of the other target genes (PCC>=0.5). Thus we ensured both the TF-target correlations and the overall inter-correlations among the targets. We next determined the differential regulation of TF and targets in cancer versus normal tissue. A 2-fold expression change threshold (i.e. log2fc=1) and paired Student’s T test were used to determine up- and down-regulated genes. The Benjamini-Hochberg method was used to control the overall false discovery rates (FDR=0.1). All datasets were analyzed with these same threshold settings. For the TFs, we only required them to be weakly differentially expressed in cancer versus normal (log2fc>=0.3 and *p*<=0.05), as we noticed some TFs may not be very strongly deregulated at the mRNA level. Then, for each TF, we counted the number of its target genes that were up- and down-regulated in cancers (‘n_up’ and ‘n_down’, respectively), and classified the TF-targets group as ‘up’ if the TF was overexpressed and n_up/n_down>=10 (vice versa).

### Gene Ontology analysis

Gene Ontology (GO) annotations for human were obtained from the org.Hs.eg.db package (Bioconductor). The GO hierarchy was downloaded from the GO official website (http://geneontology.org) and we focused on the ‘biological processes’ category, which are more relevant to functional enrichment analysis. Fisher’s exact test was used to assess the enrichment for each GO term, and those significant terms (*p*<0.05 and OR>2) were further filtered according to the GO hierarchy with a priority given to more specific terms.

## Results

### An OC-SVM model for predicting transcriptional regulatory network

To unravel the TF regulatory network in the major lung cancer subtypes, we designed a two-step strategy: first build an overall TF regulatory network, and then combine dataset information to identify dataset-specific TFs and regulation. Over the years, experimentally validated TF-target relationships have accumulated and become a valuable resource for learning general principles that would guide further discoveries of novel regulation [30–32]. For such experimental knowledge, the positive training datasets are of high quality whereas the negative datasets are mostly unavailable. To build a global TF regulatory network based on the resource available, we took advantage of an OC-SVM framework that has been widely used in the single-class prediction field [33].

**Fig. 1:**
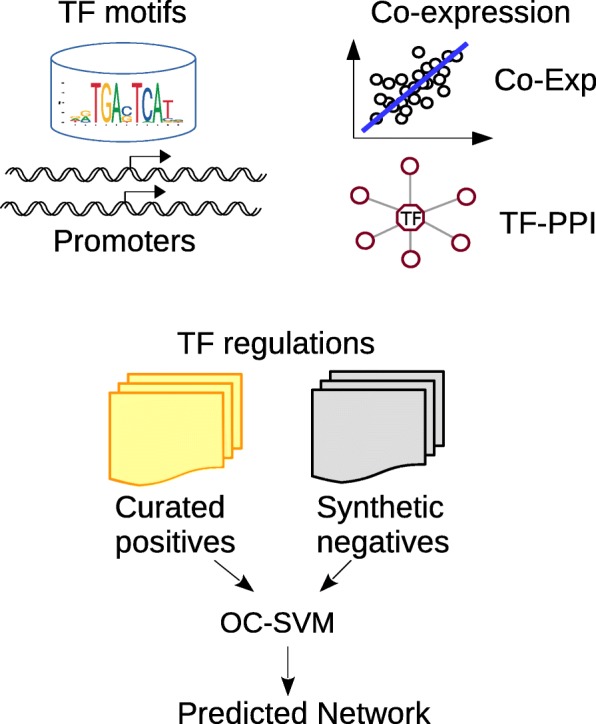
Prediction of TF targets with OC-SVM. TF binding motifs were scanned along promoter regions (-10kb~+10kb around TSS) for annotated genes. Co-expression between TF and candidate targets, as well as between the TF PPI neighborhood and candidate targets, were analyzed. An OC-SVM model was trained with curated TF-target knowledge, and synthetic negatives were used for evaluating its performance

We collected and extracted the following information for establishing TF-target relationships: the presence and distribution of TF binding motifs along the promoter regions, the co-expression between a TF and its target genes, as well as the co-expression of a TF’s interacting proteins (‘neighborhood’) with its target genes (Fig. 1, Methods).

**Fig. 2:**
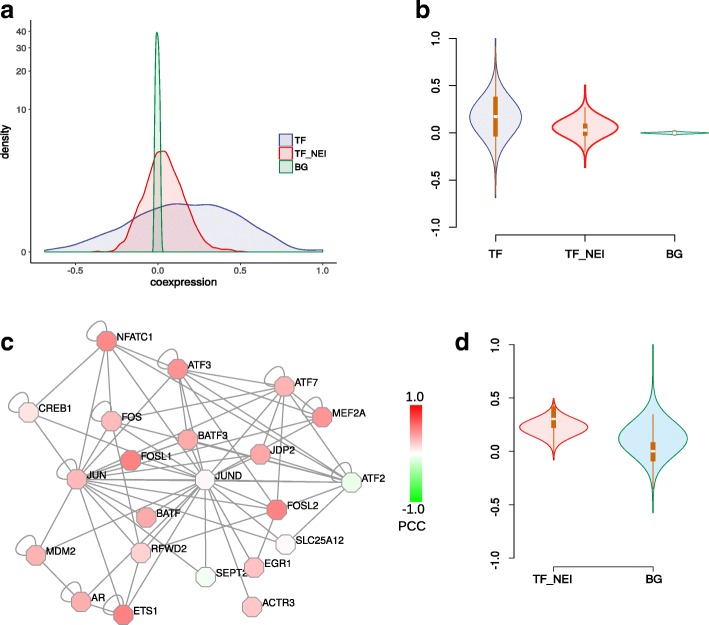
Co-expression analyses for TF, TF neighborhood and known target genes. **a**, **b** Distribution of PCCs between TFs and target genes, between TF neighborhoods and target genes, and among all genes as the background. **c** JUND and its neighborhood network. Nodes were colored according to co-expression with JUND’s known target GADD45A. **d** Co-expression distribution between JUND’s neighborhood and GADD45A

From the distribution of Pearson correlation coefficients (PCCs), there was much stronger positive co-expression than the background (Fig. 2a, b), implicating the rationality of co-expression-based TF-target prediction. In addition, the TF-interacting proteins displayed a positive but weaker co-expression with target genes. An interesting example was JUND, which regulated downstream target gene GADD45A (Fig. 2c-d, Additional file 1: Figure S1). Although JUND itself did not show clear co-expression with GADD45A, its interacting proteins indeed showed strong positive co-expression with GADD45A. Therefore we integrated the neighborhood co-expression with target genes into the OC-SVM model.

**Fig. 3:**
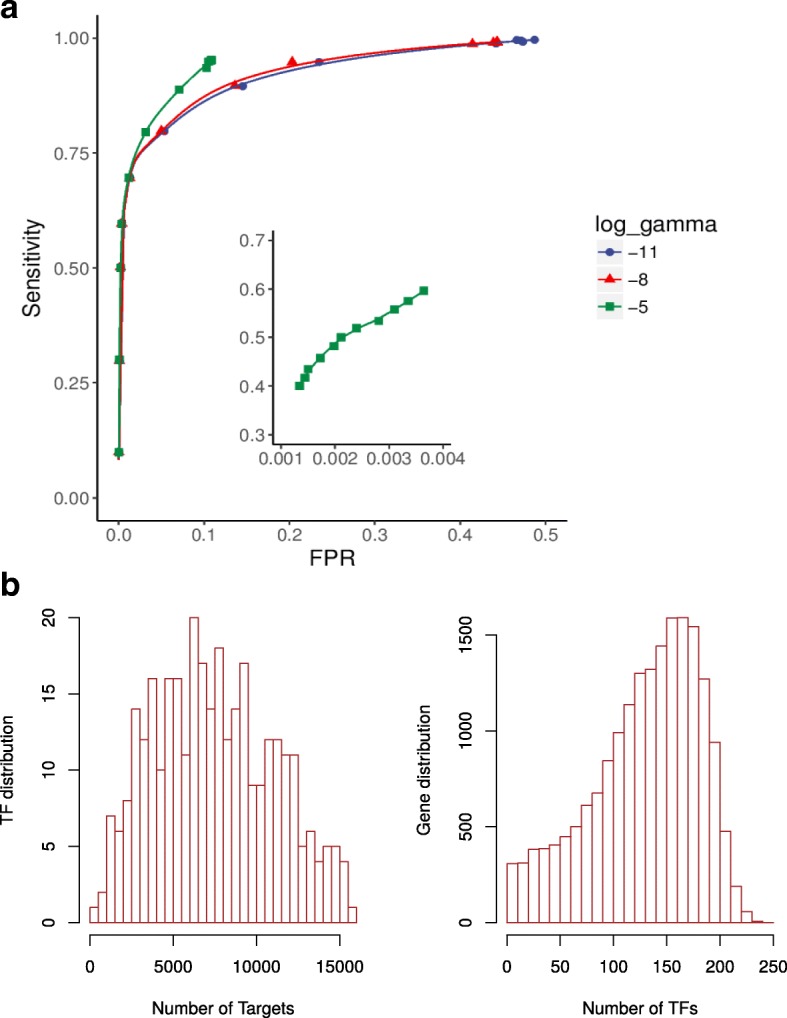
Training and prediction of the OC-SVM model. **a** ROC curves for model evaluation with 10-fold cross validation. The positive sets were curated known TF-target regulatory relationships, whereas the negative sets were artificially synthesized (See Methods). ROC curves for three values of log2 gamma parameter were shown: -11, -8, -5. **b** Predictions of OC-SVM. Left, distribution of TFs by the number of predicted targets. Right, distribution of genes by the number of TFs predicted to target them

To assess the performance of the OC-SVM model, we artificially synthesized some negative sets based on the following principles: 1) the synthetic genes’ promoter regions are randomly generated and then summarized for individual TF-binding motifs; 2) the co-expression between synthetic genes and other genes including TFs and TF neighbors were randomly extracted from real co-expression data using a randomized gene label. Model performance was evaluated with 10-fold cross-validation. At a sensitivity level of 75%, the true positive rates are generally above 90% (Fig. 3a). We realized that minimizing the FPR was critical for our tasks, since the number of possible regulatory relationships are rather huge: e.g. for 300 TFs and 20,000 genes, there would be 6 million possible relations. Therefore we had to minimize FPR as long as the sensitivity was acceptable. To further guarantee the appropriate choice of model parameters, we evaluated different parameter combinations (nu=0.3, 0.5, 0.7; log2gamma=-5, -8, -11) for TF network training, with a real dataset (TCGA LUSC) and two known core LUSC TFs (TP63 and SOX2) serving as positive controls. Each combination successfully recalled both TFs, indicating that core TFs might be identified even with a less sensitive model (Additional file 2: Table S3). Nonetheless, the number of targets predicted for each TF decreased with lower model sensitivities, emphasizing that a higher model sensitivity might be more powerful to detect core TFs (Additional file 2: Table S3). Based on the cross-validation and real dataset evaluations above, we chose an appropriate parameter combination (nu=0.5 and log2gamma=-5) to balance our specific requirements of sensitivity (~50%) and FPR (~0.2%). This resulted in a predicted network of 325 TFs and 18724 protein-coding target genes (Fig. 3b). The numbers of target genes for TFs are 7332 in median (ranging from 338 to 15929), and the numbers of regulatory TFs for genes are 139 in median (ranging from 0 to 244), indicating the network was quite general and should be narrowed down for identification of condition-specific regulation.

### Identification of dataset-specific differential transcriptional regulation

To identify condition-specific regulation, we enforced three requirements (Methods): (i) co-expression between TF and predicted targets; (ii) co-expression among the predicted targets; (iii) differential regulation between cancer and normal tissue: the TF itself should at least be weakly deregulated and its targets should be distributed in the same direction as the TF, with an enrichment of 10 fold versus the opposite direction (Methods).

In order to evaluate the effect of differential criteria on TF identification, various combinations of log2fc and FDR q value thresholds were tried on the TCGA LUSC dataset. Although the numbers of up- and down-regulated genes fluctuated greatly, the TFs identified were quite stable, indicating the robustness of the methodology (Additional file 2: Table S4). Therefore, the same differential threshold (|log2fc|>=1 and q<=0.1) was applied to all datasets.

We applied the above analyses and requirements to the following lung cancer datasets (Methods), and identified dataset-specific regulatory TFs: TCGA LUAD (referred to as ‘LUAD’), TCGA LUSC (referred to as ‘LUSC’), SCLC dataset (referred to as ‘SCLC’), independent LUAD and LUSC dataset (referred to as ‘LUAD2’ and ‘LUSC2’ respectively) (Additional file 2: Table S1). We also clustered the up- and down-regulated TFs according to their targets overlapping to identify potential co-regulated TFs (Fisher’s exact test, *p* < 0.05).

### The TP63/SOX2/DMRT3 circuit as a hallmark of lung squamous carcinomas

**Fig. 4:**
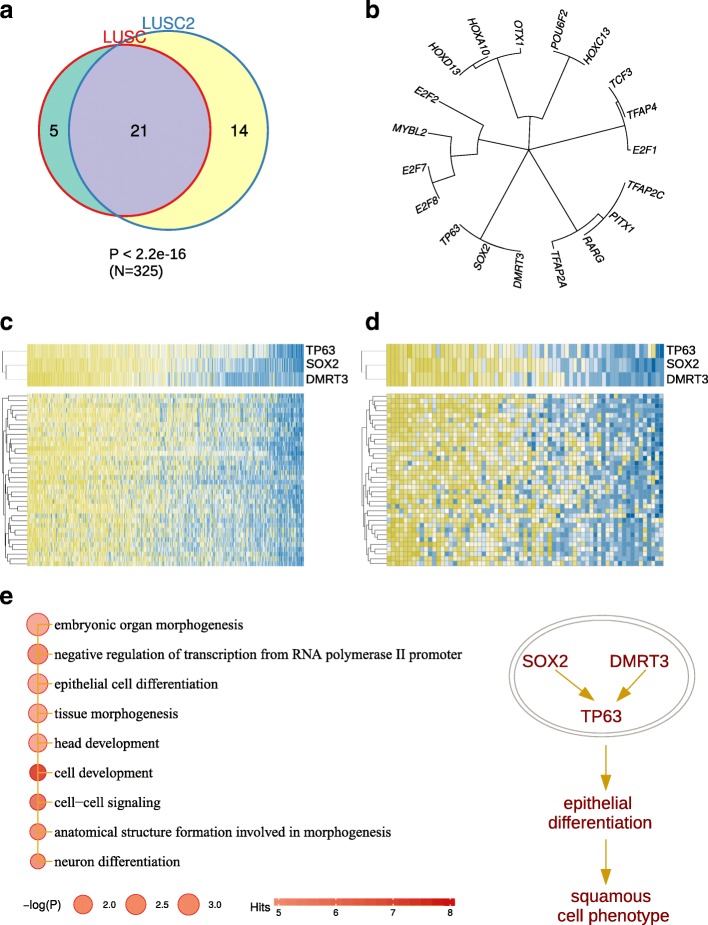
Transcriptional hallmarks for LUSC. **a** Consistency of up-regulated TFs identified in the LUSC and LUSC2 datasets. **b** Clustering of up-regulated TFs shared in the two LUSC datasets. TFs with 10 or fewer targets shared between the two datasets have been filtered out before clustering. Cluster membership was determined using Fisher’s exact test (*p*<0.05). **c**, **d** Expression patterns of the TP63/SOX2/DMRT3 module and their commonly regulated genes in LUSC (**c**) and LUSC2 (**d**) datasets. **e** Functional enrichment of co-regulated genes by TP63/SOX2/DMRT3 (left). A hypothetical regulatory model was proposed (right)

We identified 26 up-regulated TFs in LUSC, 21 of which were also identified in the LUSC2 dataset independently, suggesting a good agreement between different datasets (Fig. 4a, Additional file 3: Figure S2A, Additional file 2: Table S1). We then merged these two sets of up-regulated TFs and only retained those with shared target genes. A further clustering of these TFs showed some of them were well clustered into TF modules (Fig. 4b, Additional file 3: Figure S2B).

Among these, TP63 and SOX2 were well-known LUSC-specific oncogenic TFs that were important in squamous epithelial differentiation and/or survival [15–17, 34–36]. Moreover, our analyses indicated that DMRT3 was associated with TP63 and SOX2 in the same module (Fig. 4b-d). The functional implication of DMRT3 in LUSC was not well known, though two earlier studies found that DMRT3 could be lost through copy number alteration mechanisms in LUSC [37, 38]. To reconcile this seeming discrepancy, we exploited inter-correlations among DMRT3 copy number, DMRT3 expression, and TP63/SOX2 expression through an integrative analyses of the TCGA data. We found that the copy number status of DMRT3 was heterogeneous in LUSC, with tumors not bearing DMRT3 deletions having significantly higher DMRT3 expression, as well as significantly increased TP63/SOX2 expression (Additional file 3: Figure S2C-E). These indicated that DMRT3 might have dual functions correlated with the heterogeneity of LUSC, with its higher expression mainly restricted to samples overexpressing TP63/SOX2. In addition, both SOX2 and DMRT3 targeted the TP63 promoter (Additional file 3: Figure S2F), and these three factors altogether co-regulated a common subset of genes involved in epithelial cell differentiation (Fig. 4e, left). Therefore, we hypothesize that DMRT3 may participate in the TP63/SOX2 circuit for regulating squamous cell differentiation and/or survival, and that these three factors may co-regulate genes functioning in human LUSC development and squamous phenotype formation (Fig. 4e, right). Interestingly, a more recent study identified DMRT3 as an important regulator of neuronal differentiation programs involved in locomotor network development [39]. Future experimental studies are worth to fully characterize the implication of DMRT3 with SOX2/TP63 in augmenting LUSC epithelial survival.

Furthermore, a comparison with the other two lung cancer subtypes revealed that, the TP63/SOX2/DMRT3 circuit was among the TFs up-regulated in a LUSC-specific manner (Fig. 7c), consistent with known properties of squamous lineage survival TFs.

### Functional regulation transcriptionally encoded in lung adenocarcinomas

**Fig. 5:**
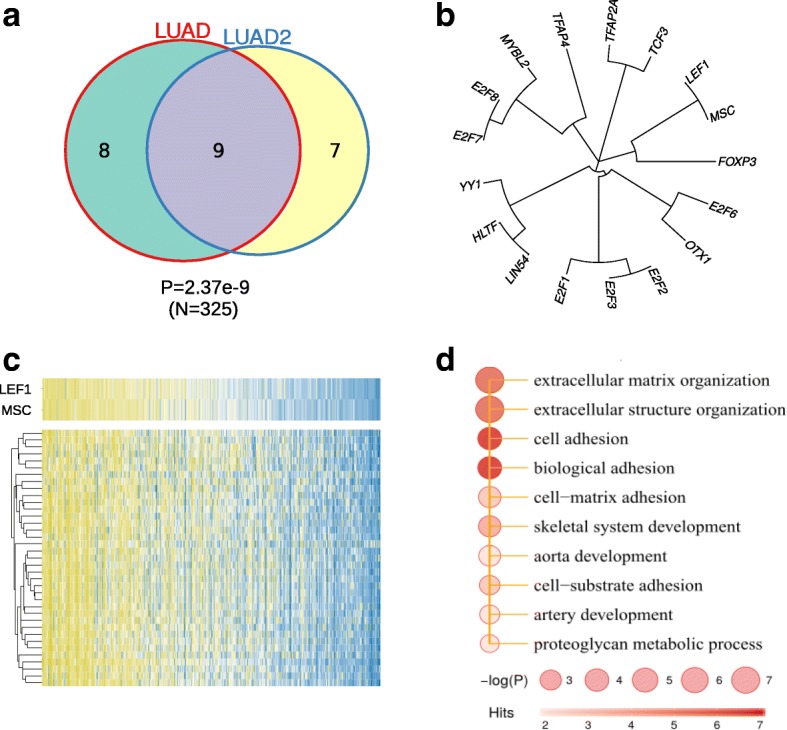
Transcriptional deregulation in LUAD. **a** Consistency of up-regulated TFs identified in the LUAD and LUAD2 datasets. **b** Clustering of up-regulated TFs identified in the TCGA LUAD dataset. Cluster membership was determined using Fisher’s exact test (*p*<0.05). **c** Expression pattern of the LEF1/MSC module and their common targets in TCGA LUAD dataset. **d** Functional enrichment of genes co-regulated by LEF1/MSC

We next analyzed the TF modules that were up-regulated in LUAD (Fig. 5). The two independent datasets again show good agreement, although not as good as that in LUSC datasets (Fig. 5a). To reduce batch effects, we restricted our analyses to the LUAD dataset. Several LUAD TFs were commonly shared with LUSC, such as E2F7, E2F8, MYBL2, TFAP2A, TFAP4 and OTX1 (Fig. 4b, 5b, Additional file 2: Table S1). Other TFs such as LEF1 (Lymphoid Enhancer-binding Factor 1) and MSC (Musculin, also Activated B-Cell Factor 1) were specific to LUAD and not present in LUSC or SCLC (Fig. 7c, Additional file 2: Table S1). LEF1 is in the Wnt signaling pathway and known to regulate the EMT process. It has been found to be activated in multiple cancer types ranging from leukemia to solid tumors including LUAD [40]. Consistent with its function in EMT, LEF1 drives metastasis of primary LUAD to brain and bone [41]. The other factor, MSC, is less studied in lung cancer. Nonetheless, its overexpression has been implicated in disruption of normal B cell differentiation program and Hodgkin lymphoma development [42]. These data suggest that MSC and LEF1 might functionally converge at EMT. In LUAD, MSC and LEF1 clustered together to regulate a shared set of target genes (Fig. 5b). Furthermore, analyses of these genes co-regulated by MSC and LEF1 revealed significant enrichment of terms such as extracellular matrix (ECM) organization and cell-ECM interactions, which were related to EMT (Fig. 5c, d). Together, our data showed that two LUAD-specific TFs, MSC and LEF1, might synergize in promotion of lung cancer malignant progression through EMT process.

Surprisingly, NKX2-1, a TF amplified in about 12% of LUAD [43], turned out to be a down-regulated regulator in the TCGA LUAD dataset, and not identified in the LUAD2 dataset (Additional file 4: Figure S3B, Additional file 5: Figure S4, Additional file 2: Table S1). Several observations might help explain this unexpected result. First, NKX2-1 was amplified in only a limited subset of LUAD tumors (Additional file 4: Figure S3C) [43]. Second, NKX2-1 expression showed a stage-dependent manner, with up-regulation in stage I and gradual down-regulation from stage II to IV (Additional file 4: Figure S3D), in consistent with previous publication [44]. Third, it has been proposed that NKX2-1 plays dual roles in LUAD, both oncogenic and anti-oncogenic (also anti-metastatic) in LUAD [45, 46]. Taken together, NKX2-1 may have stage-specific function in LUAD and tends to be down-regulated as LUAD become advanced.

### Regulatory patterns specific to small-cell lung carcinomas

**Fig. 6:**
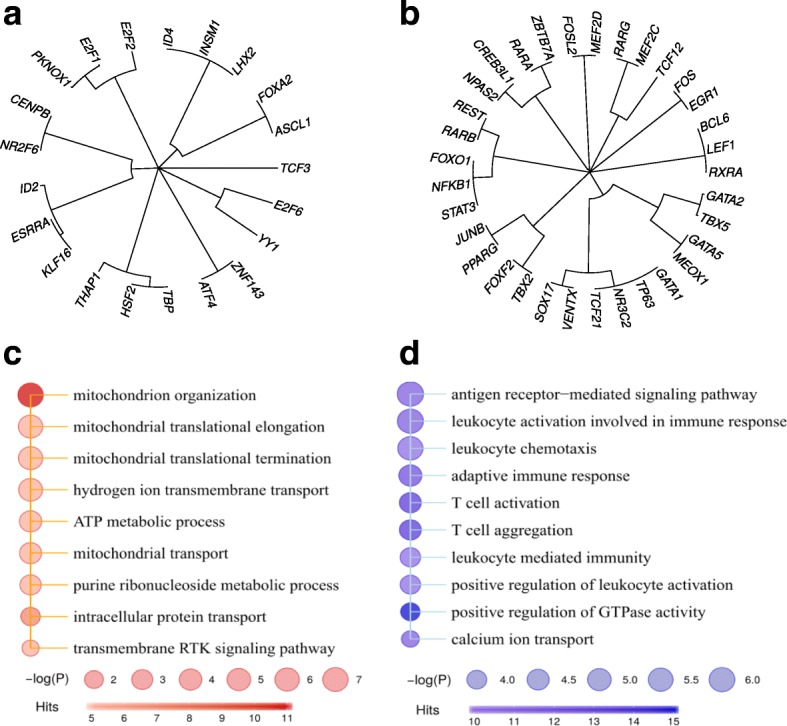
Transcriptional deregulation in SCLC. **a**-**b** Clustering of up-regulated (**a**) and down-regulated (**b**) TFs, respectively. Cluster membership was determined using Fisher’s exact test (*p*<0.05). **c** Functional enrichment of ID2 target genes in SCLC. **d** Functional enrichment of ID2 target genes in LUSC

**Fig. 7:**
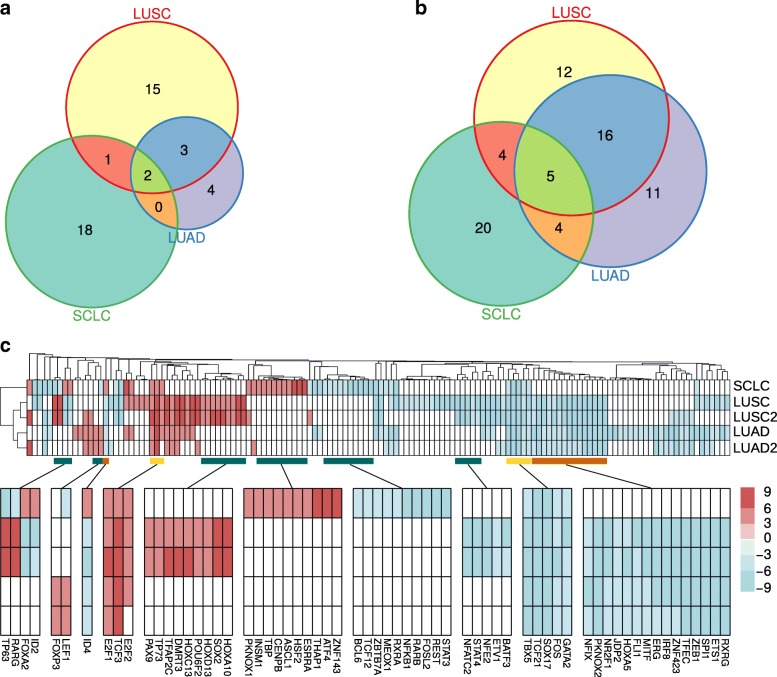
Landscape of transcriptional deregulation in lung cancer. **a** Comparison of up-regulated TFs in LUAD, LUSC and SCLC datasets. **b** Comparison of down-regulated TFs in LUAD, LUSC and SCLC datasets. **c** The global patterns of TF deregulation across the five datasets: LUAD, LUAD2, LUSC, LUSC2 and SCLC. Colors reflect the log2 scaled number of a TF’s targets, with up-regulated TFs in red and down-regulated in blue. Selected branches of TFs that were common (orange for NSCLC-common, yellow for all-common) or subtype-specific (blue) are highlighted (bottom)

Traditionally, LUAD and LUSC are categorized in the NSCLC group, as SCLC is distinct in its cell size, shape and cell mitosis rate. In SCLC, we found those uniquely up-regulated TFs such as ASCL1, CENPB, HSF2, ZNF143 and down-regulated TFs such as STAT3, REST, NFKB1, different from those in LUAD and LUSC (Fig. 6a-b, Fig. 7c, Additional file 2: Table S1). Among these, the bHLH family TF ASCL1, a well-known neuronal differentiation regulator, is required by neuroendocrine tumors including SCLC [47–49]. ASCL1 target genes showed an involvement in regulation of neurotransmitter levels and presynaptic process related to synaptic transmission (Additional file 2: Table S2). Moreover, the target genes of ASCL1 were significantly shared by FOXA2, whose target genes were also enriched for neural-related functions including neuronal generation and cell migration (Additional file 2: Table S2). These again emphasized the unique neuroendocrine features of SCLC, in contrast to LUAD and LUSC.

Interestingly, some TFs showed opposite expression changes in comparison with LUAD and/or LUSC. For example, ID2, FOXA2 and ID4 were up-regulated in SCLC but down-regulated in LUAD and/or LUSC. Similarly, TP63 and RARG were down-regulated in SCLC but up-regulated in LUSC (Fig. 7c). We next explored the potentially opposite roles of ID2 in SCLC and LUSC. In SCLC, ID2 regulates mitochondrion organization, mitochondrion protein translations and ATP synthesis (Fig. 6c), and its up-regulation probably assisted SCLC cells in gaining sufficient energy to support fast mitosis and proliferation. However, in LUSC, ID2 conditionally regulated another set of genes involved in positive regulation of immune response, leukocyte cell activation and immune signaling (Fig. 6d), and down-regulation of ID2 and its target genes help LUSC cells to escape immune surveillance. This indicated that different types of cancer cells may deregulate the same TF differently, in support of cancer-specific need in malignant progression.

### The transcriptional regulatory landscape of lung cancer subtypes

We have unraveled the key TFs as well as their targets in each of the three major subtypes of lung cancer (Fig. 7c, Additional file 5: Figure S4, Additional file 2: Table S1). Notably, there were some deregulated TFs shared by all three subtypes. For example, two TFs, E2F1 and TCF3, were up-regulated in all three subtypes (Fig. 7a, c). These two factors both regulated target genes mainly involved in cell cycle and/or cell division processes (Additional file 2: Table S2). We found that E2F1 regulated genes enriched in ‘cell division’ across all three subtypes, with three target genes in the GO term commonly regulated in lung cancers: CCNF (cyclin F), NCAPH (Non-SMC Condensin I Complex Subunit H), SPAG5 (Sperm Associated Antigen 5). Moreover, five TFs were found to be down-regulated in all three subtypes: FOS, GATA2, SOX17, TBX5, TCF21 (Fig. 7b, c). They regulate various functions ranging from ‘inflammatory response’ to ‘positive regulation of apoptotic process’. Some TFs shared the same target genes across the different subtypes, e.g., FLI1 probably targets CCRL2 (Chemokine/C-C Motif Receptor-Like 2), an essential regulator of leukocyte recruitment in the lung [50], in all three subtypes.

We also found dramatic difference of regulation patterns among the subtypes. The two NSCLC isoforms (LUAD and LUSC) shared more TFs than with SCLC (Fig. 7a, b). LUAD and LUSC shared 5 up-regulated (TFAP4, OTX1, E2F8, E2F1, TCF3) and 21 down-regulated factors (ID4, RXRG, JDP2, MITF, SPI1, NFIX, NR2F1, ZEB1, ZNF423, ERG, TFEC, ETS1, HOXA5, PKNOX2, TCF21, FLI1, SOX17, TBX5, IRF8, FOS, GATA2). The up-regulated TFs mainly regulated cell proliferation (‘mitotic nuclear division’, ‘cell division’, ‘G1/S transition of mitotic cell cycle’ and ‘DNA repair’), and the down-regulated TFs mainly regulated cell differentiation (‘mesenchymal cell differentiation’, ‘lung development’, ‘embryonic morphogenesis’, ‘pattern specification process’), cell proliferation (‘negative regulation of cell proliferation’) and immune responses (‘inflammatory response’, ‘T cell proliferation’, ‘T cell aggregation’) (Additional file 2: Table S2). SCLC specifically up-regulated a series of TFs (ASCL1, FOXA2, ID2, ID4, THAP1, ATF4, CENPB, ZNF143, HSF2, ESRRA, TBP, INSM1, PKNOX1) that functioned in neural functions (‘regulation of neurotransmitter levels’, ‘presynaptic process’, ‘generation of neurons’, ‘neuron development’, ‘neurological system process’), mitochondrial activities (‘mitochondrion organization’, ‘mitochondrial translational elongation’), protein synthesis (‘translation’, ‘rRNA processing’), metabolism (‘purine ribonucleoside metabolic process’) and cell proliferation (‘mitotic cell cycle process’, ‘cell division’). Those down-regulated TFs in SCLC (JUNB, NFKB1, VENTX, CREB3L1, REST, RARB, FOXO1, EGR1, TP63, ZBTB7A, STAT3, MEOX1, FOSL2, RARG, GATA5, RXRA, NPAS2, LEF1, BCL6, TCF12) were functionally linked to cell differentiation (‘positive regulation of cell differentiation’, ‘epithelial cell differentiation’) and immune responses (‘inflammatory response’, ‘T cell aggregation’, ‘positive regulation of cytokine production, ‘leukocyte migration’) (Additional file 2: Table S2). These findings indicated that NSCLC and SCLC hijacked different molecular machineries to promote malignant progression. Nonetheless, SCLC had more specific TF circuits to increase mitochondrial activities and protein synthesis, which probably provided high levels of cellular energy in support of fast mitosis [51].

A notable difference of TF circuits was even detected between LUAD and LUSC, two major subtypes of NSCLC. LUAD specifically up-regulated several TFs (LEF1, E2F3, HLTF, FOXP3), whereas LUSC preferentially up-regulated other TFs (SOX2, TP63, DMRT3, PITX1, E2F7, TFAP2A, MYBL2, HOXA10, HOXC13, RARG, TFAP2C, POU6F2, HOXD13, PAX9, TP73, E2F2). Besides the common function enriched for these two up-regulated sets of LUAD- and LUSC-specific TFs (‘mitotic nuclear division’, ‘cell proliferation’), there were unique functions enriched for LUSC (‘epithelial cell differentiation’, ‘epidermis development’, ‘skin development’) (Additional file 2: Table S2), and the TP63/SOX2/DMRT3 cluster was closely related to this squamous differentiation program.

## Discussion

Transcriptional regulation serves as the fundamental regulatory program in orchestrating normal development and disease progression. To unravel the transcriptional target genes of TFs, both experimental techniques (e.g. SELEX, ChIP-on-chip, ChIP-seq) and computational methods have been successfully developed. Traditionally, TF binding preferences can be characterized as position-weight matrices (PWMs), which are then used to scan the promoter regions for potential hits. Although PWM-based methods and extensions have been widely followed and deeply exploited [52–59], sequence-based methods per se are not sufficient to account for the full TF-DNA interaction specificities in vivo [60, 61]. To enhance the specificity of target gene predictions, it is useful to incorporate expression relevance between TF and targets [62, 63]. However, as TFs may often be regulated by post-translational modifications, translocations, as well as protein-protein interactions, its expression level could not fully represent the regulatory activity. To remedy this, we used a network-based approach to incorporate expression relevance dispersed in the TF neighborhood. Through the integration of PWM matching, expression correlations, and neighborhood relevance, an OC-SVM model was trained and evaluated for the performance in predicting known targets, which allowed us to control the false discovery rate to 0.002.

Another major motivation of this work is to present the landscape of transcriptional deregulation of lung cancer including three major subtypes LUAD, LUSC and SCLC. We reveal those common regulatory relationships as well as subtype-specific regulatory relationships. We have distinguished up- and down-regulation of TF circuits in each subtype, and predicted a number of subtype-specific TF modules (e.g. TP63/SOX2/DMRT3, LEF1/MSC, ASCL1 and ID2). Moreover, we have interpreted each module to functionally explain that different mechanisms are hijacked by different cancer cells to achieve corresponding malignant progression. Notably, many of these functional outputs are highly correlated, such as cell proliferation, dedifferentiation and immune suppression. Nonetheless, different subtypes of lung cancer also harbor unique TF machinery in contribution to tumor growth. For example, in SCLC, many unique TF circuits are related to mitosis, protein synthesis, mitochondrial activities and energetic metabolism, which are certainly important for promoting fast cell division. The epithelial differentiation programs are also dramatically elevated in LUSC, which are known important for squamous cell lineage survival from studies of cell lines and mouse models.

There are also some limitations of this study. We have not necessarily required a TF itself to be co-expressed with its target genes when training the general regulatory network. However, during the dataset analyses, we still require the TF to have at least weak expression changes (through using less stringent thresholds), as we want to focus on those TFs that can be regulated at expression level, which is also common for many TFs important in the regulation of differentiation. Nonetheless, this may miss some TFs that are transiently regulated without long-term changes in expression. In addition, we restrict our analyses to activating TFs that up-regulate target genes, but the number of TFs that are repressive is also nonnegligible. Future work will be needed to integrate them into a more flexible model. Moreover, the SCLC dataset that we used lacks normal controls, and so we used the adjacent normal samples in the LUAD and LUSC datasets to compare with SCLC. Although those adjacent normal tissues from LUAD and LUSC are quite similar (Additional file 6: Figure S5), we cannot rule out the possibility that those from SCLC might be different.

The complete landscape of complex deregulation in various lung cancer subtypes still contains many gaps and missing parts. This work provides an initial comprehensive study to unravel the overall patterns with an emphasis on those important circuits in lung cancer. Future studies from both computational and experimental approaches would be necessary to decode and validate the transcriptional networks in various lung cancer subtypes, including those not covered here, such as LCC.

## Conclusions

We have systematically studied the core transcriptional deregulation in three well-characterized lung cancer subtypes (LUAD, LUSC and SCLC), and identified a number of common (e.g. proliferation-related E2F1 and TCF3) as well as subtype-specific TF circuits (e.g. the epithelial-development-related TP63/SOX2/DMRT3 module in LUSC, the EMT-related LEF1/MSC module in LUAD, and the neural differentiation regulator ASCL1 in SCLC). Moreover, ID2 targets two different sets of genes with one involved in mitochondrial activities in SCLC and the other involved in immune response in LUSC, highlighting the importance of the same TF differentially regulated in different cancer subtypes. Nonetheless, different TFs are also employed by NSCLC and SCLC to achieve similar functional consequences to support tumor progression.

## Additional files

Additional file 1: **Figure S1.** Co-expression between JUND or its neighborhood and its known target gene GADD45A. Three of JUND’s neighborhood genes with strongest co-expression with GADD45 were chosen for display. (PDF 93 kb)

Additional file 2: **Table S1.** TFs deregulated in each lung cancer dataset. Columns are: DS (dataset), DIR (direction of regulation), TF, lfc (log2 fold change), p (differential t test p value), ntargs (number of targets deregulated) and targs (targets deregulated). **Table S2.** GO terms enriched in targets of each TF. Columns are: DS (dataset), DIR (direction of regulation), TF, GO, Term, Annotated (Number of genes annotated and recognized in GO term), GOI (Number of deregulated targets for each TF), Hits (TF targets annotated in the GO term), OR (odds ratio), pFisher (Fisher’s Exact Test p value) and Genes (Gene Hits). **Table S3.** Evaluation of SVM parameters. Different parameter combinations were used to set up the OC-SVM model for training. Each model was used to predict a TF network, which was then applied to the LUSC dataset to see if the two positive control TFs (TP63 and SOX2) can be recalled. **Table S4.** Evaluation of differential analysis parameters. Several combinations of log2fc and q value thresholds were applied to determine up- and down-regulated genes in the LUSC dataset, which are further used for TF identification. The identified TFs are compared with each other to evaluate the procedural robustness upon the various parameter choices. Top left and right: summarizing tables; Bottom: detailed tables of TFs identified in each parameter combination. (XLS 3594 kb)

Additional file 3: **Figure S2.** Down-regulation of TFs in LUSC. (A) Consistency of down-regulated TFs identified in the LUSC and LUSC2 datasets. (B) Clustering of down-regulated TFs shared in the two LUSC datasets. Cluster membership was determined using Fisher’s exact test (*p*<0.05). (C) DMRT3 expression grouped by DMRT3 copy number status (deletion vs. non-deletion) (Wilcoxon signed-rank test). (D) DMRT3 loss status in relation to TP63 expression (Wilcoxon signed-rank test). (E) DMRT3 loss status in relation to SOX2 expression (Wilcoxon signed-rank test). (F) SOX2 (red) and DMRT3 (blue) binding motifs on the TP63 promoter (-10kb to +10kb of TSS). Genomic coordinates are according to the hg19 assembly. (PDF 92 kb)

Additional file 4: **Figure S3.** Down-regulation of TFs in LUAD. (A) Consistency of down-regulated TFs identified in the LUAD and LUAD2 datasets. (B) Clustering of down-regulated TFs identified in the TCGA LUAD dataset. Cluster membership was determined using Fisher’s exact test (*p*<0.05). (C) NKX2-1 copy number distribution in TCGA-LUAD dataset. (D) NKX2-1 expression in normal lung and LUAD categorized by tumor stage (I to IV). (PDF 94 kb)

Additional file 5: **Figure S4.** The complete version of Fig. 7c, showing the global TF deregulation patterns across the five datasets: LUAD, LUAD2, LUSC, LUSC2 and SCLC. Colors reflected the log2 scaled number of a TF’s targets, with up-regulated TFs in red and down-regulated in blue. (PDF 22 kb)

Additional file 6: **Figure S5.** Consistency among the normal lung tissues from the four datasets: TCGA-LUAD, TCGA-LUSC, LUAD2 and LUSC2. The PC1 and PC2 axes from Principal Component Analysis (PCA) together explained 91.8% of total variance. A good consistency of these normal lung tissues justified the assumption that they could be pooled together for comparison with SCLC cancer samples. (PDF 42 kb)

## Abbreviations

CCNF: Cyclin F
CCRL2: Chemokine/C-C Motif Receptor-Like 2
ECM: Extracellular matrix
EMT: Epithelial-to-mesenchymal transition
FDR: False discovery rate
FPR: False positive rate
GO: Gene Ontology
LCC: Large-cell carcinoma
LEF1: Lymphoid Enhancer-binding Factor 1
LUAD: Lung adenocarcinoma
LUSC: Lung squamous cell carcinoma
MSC: Musculin
NCAPH: Non-SMC Condensin I Complex Subunit H
NSCLC: Non-small-cell lung carcinoma
OC-SVM: One-class support vector machine
PCC: Pearson Correlation Coefficient
PPI: Protein-protein interaction
PWM: Position-weight matrix
SCLC: Small-cell lung carcinoma
SPAG5: Sperm Associated Antigen 5
TF: Transcription factor
