# Supplementary figures and images for: Landscape of transcriptional deregulation in lung cancer
Source: BMC Genomics. 2018 Jun 5;19:435. doi: 10.1186/s12864-018-4828-1 (PMC5987572; doi:10.1186/s12864-018-4828-1)

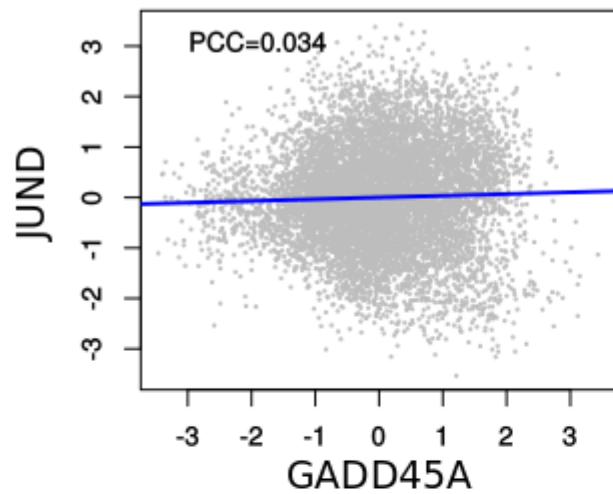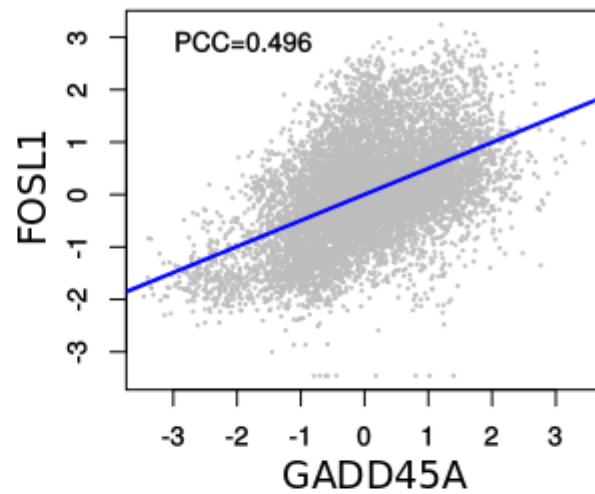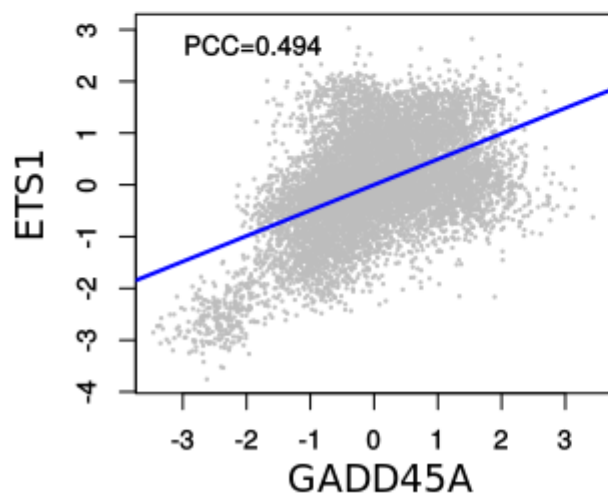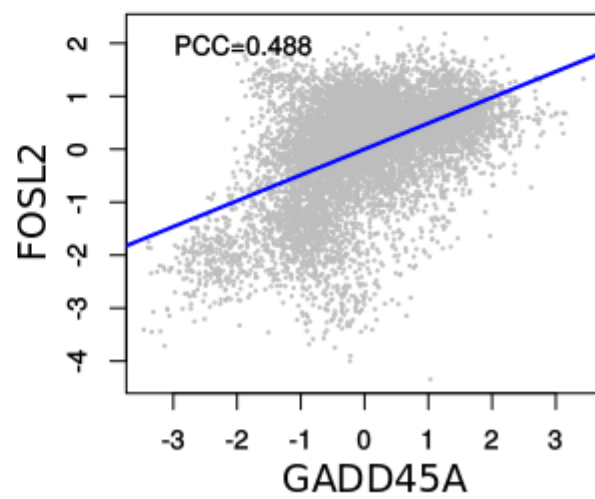

Supplement: Supplementary file 1 — Figure S1. Co-expression between JUND or its neighborhood and its known target gene GADD45A. Three of JUND’s neighborhood genes with strongest co-expression with GADD45 were chosen for display. (PDF 93 kb) [file 12864_2018_4828_MOESM1_ESM.pdf]

A

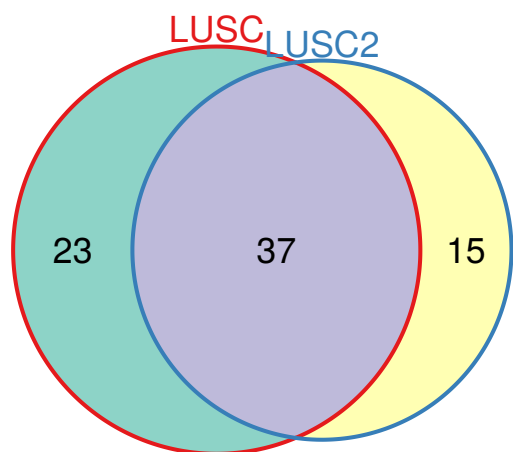

B

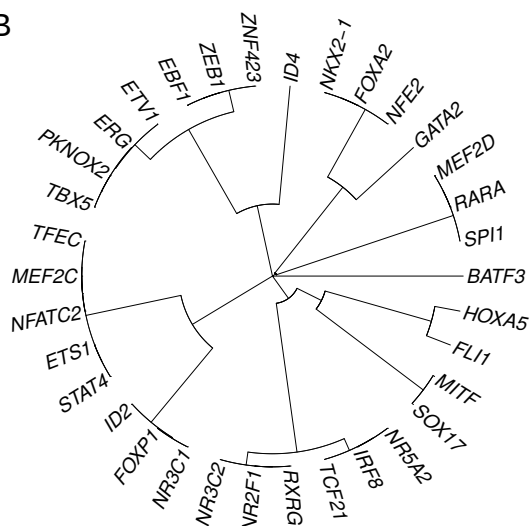

C

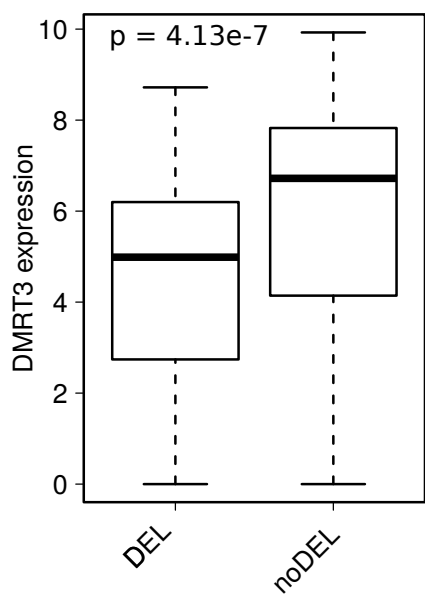

D

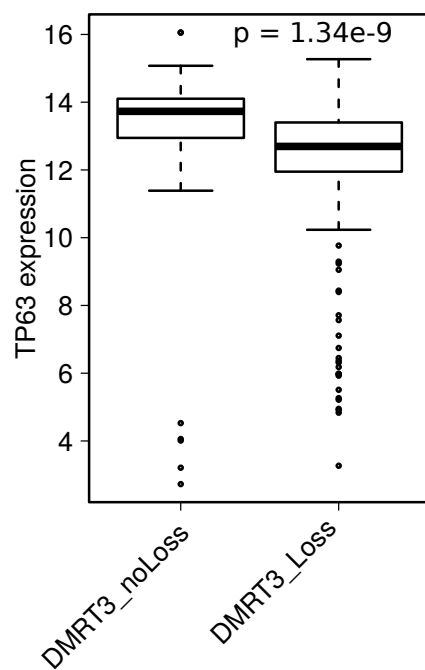

E

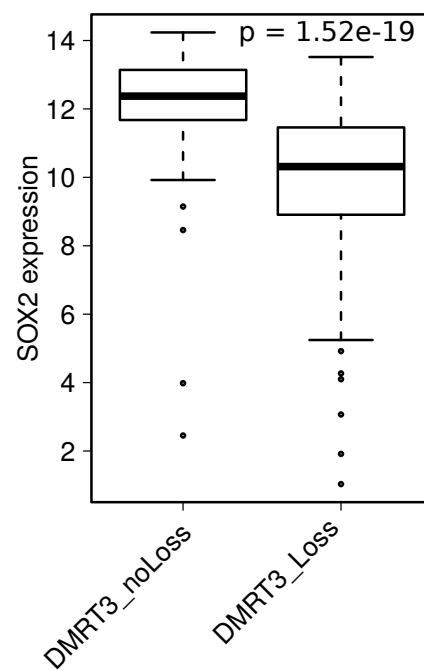

F

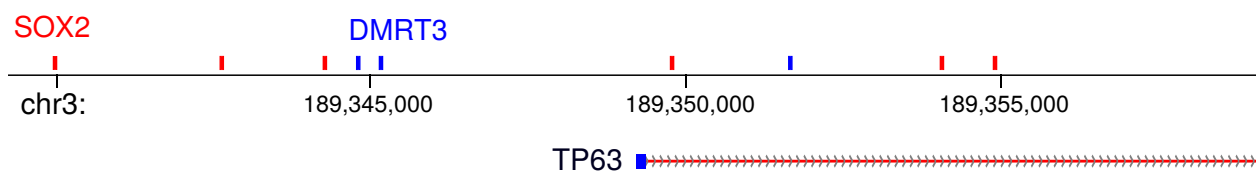

Supplement: Supplementary file 3 — Figure S2. Down-regulation of TFs in LUSC. (A) Consistency of down-regulated TFs identified in the LUSC and LUSC2 datasets. (B) Clustering of down-regulated TFs shared in the two LUSC datasets. Cluster membership was determined using Fisher’s exact test (p<0.05). (C) DMRT3 expression grouped by DMRT3 copy number status (deletion vs. non-deletion) (Wilcoxon signed-rank test). (D) DMRT3 loss status in relation to TP63 expression (Wilcoxon signed-rank test). (E) DMRT3 loss status in relation to SOX2 expression (Wilcoxon signed-rank test). (F) SOX2 (red) and DMRT3 (blue) binding motifs on the TP63 promoter (-10kb to +10kb of TSS). Genomic coordinates are according to the hg19 assembly. (PDF 92 kb) [file 12864_2018_4828_MOESM3_ESM.pdf]

A

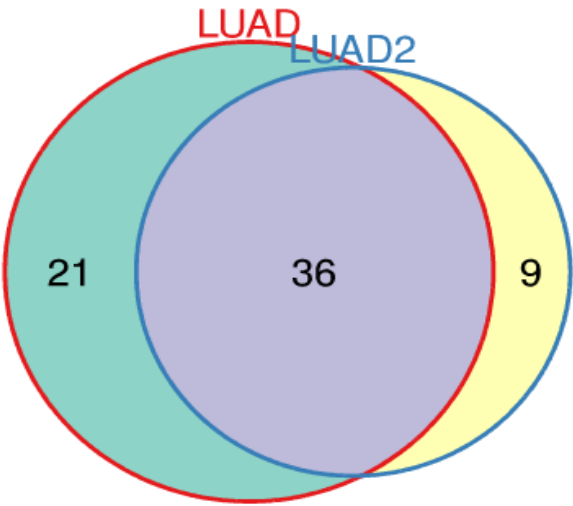

B

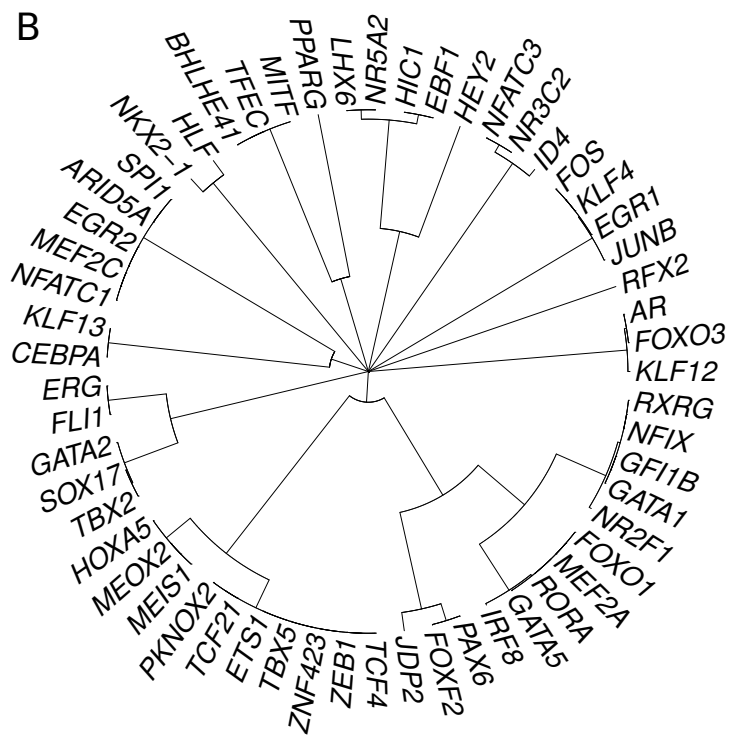

C

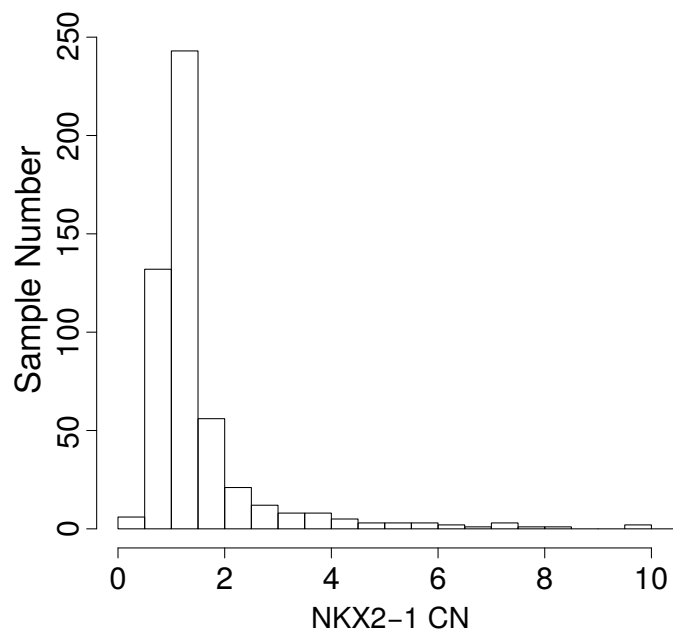

D

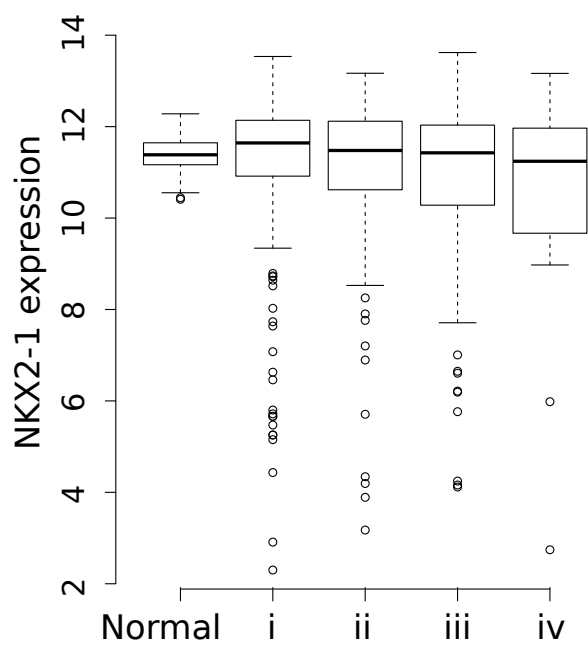

Supplement: Supplementary file 4 — Figure S3. Down-regulation of TFs in LUAD. (A) Consistency of down-regulated TFs identified in the LUAD and LUAD2 datasets. (B) Clustering of down-regulated TFs identified in the TCGA LUAD dataset. Cluster membership was determined using Fisher’s exact test (p<0.05). (C) NKX2-1 copy number distribution in TCGA-LUAD dataset. (D) NKX2-1 expression in normal lung and LUAD categorized by tumor stage (I to IV). (PDF 94 kb) [file 12864_2018_4828_MOESM4_ESM.pdf]

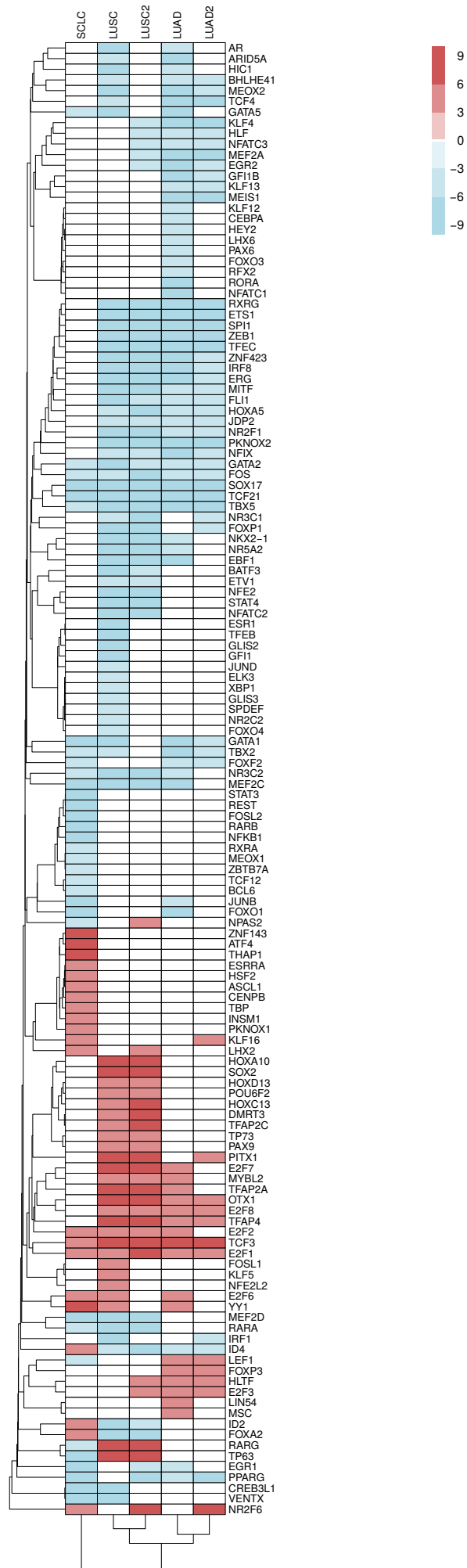

Supplement: Supplementary file 5 — Figure S4. The complete version of Fig. 7c, showing the global TF deregulation patterns across the five datasets: LUAD, LUAD2, LUSC, LUSC2 and SCLC. Colors reflected the log2 scaled number of a TF’s targets, with up-regulated TFs in red and down-regulated in blue. (PDF 22 kb) [file 12864_2018_4828_MOESM5_ESM.pdf]

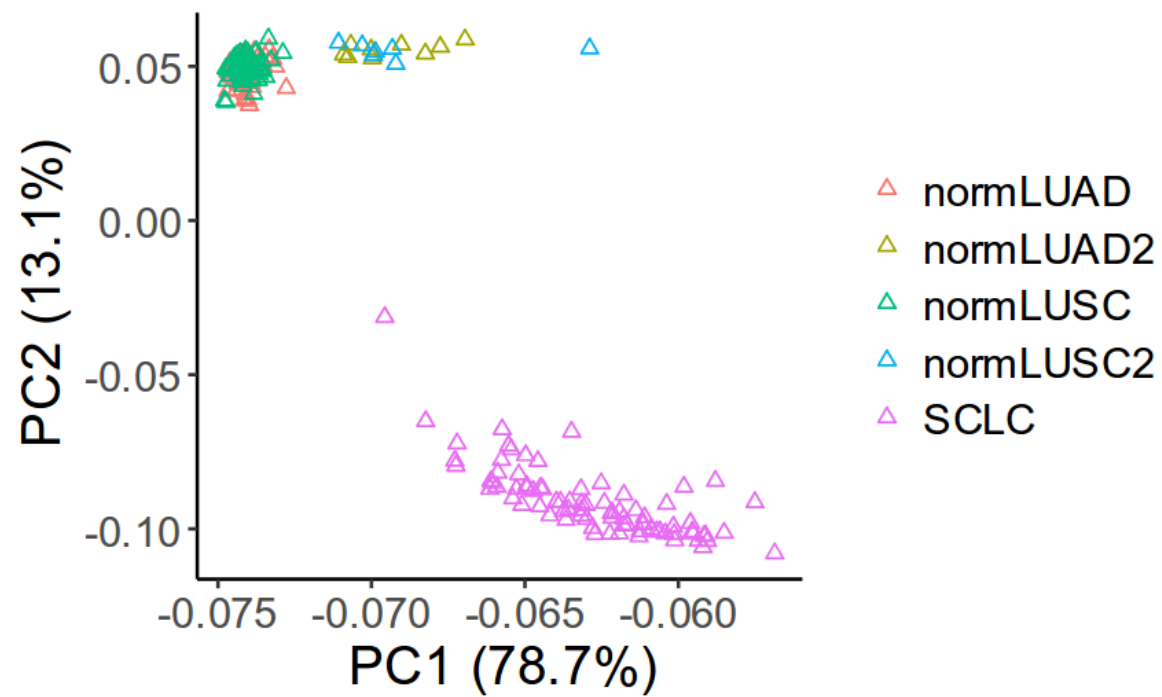

Supplement: Supplementary file 6 — Figure S5. Consistency among the normal lung tissues from the four datasets: TCGA-LUAD, TCGA-LUSC, LUAD2 and LUSC2. The PC1 and PC2 axes from Principal Component Analysis (PCA) together explained 91.8% of total variance. A good consistency of these normal lung tissues justified the assumption that they could be pooled together for comparison with SCLC cancer samples. (PDF 42 kb) [file 12864_2018_4828_MOESM6_ESM.pdf]
